# Drug-resistant tuberculosis: advances in diagnosis and management

**DOI:** 10.1097/MCP.0000000000000866

**Published:** 2022-02-25

**Authors:** Gunar Günther, Nunurai Ruswa, Peter M. Keller

**Affiliations:** a Department of Pulmonology, Inselspital, Bern University Hospital, University of Bern, Bern, Switzerland; b Department of Medical Sciences, School of Medicine, University of Namibia; c Katutura State Hospital; d National Tuberculosis and Leprosy Programme, Ministry of Health and Social Services, Windhoek, Namibia; e Institute for Infectious Diseases, University of Bern, Bern, Switzerland

**Keywords:** bedaquiline, drug-resistant tuberculosis, pretomanid, resistance prediction

## Abstract

Diagnosis and treatment of drug-resistant tuberculosis (DR-TB) is undergoing substantial changes, owing availability of new diagnostic tools and drugs, coupled with global underdiagnosis and undertreatment. Recent developments are reviewed.

**Recent findings:**

Molecular diagnostics, for *Mycobacterium tuberculosis* complex detection and prediction of drug resistance, implemented in the last decade, accelerated TB diagnosis with improved case detection. Nevertheless, access and coverage of drug-resistance testing remain insufficient. Genome sequencing-technologies, based on targeted next-generation sequencing show early potential to mitigate some of the challenges in the future. The recommendation to use an all oral, bedaquiline based regimen for treatment of multidrug-resistant/rifampicin-resistant TB is major advancement in DR-TB care. TB regimen using new and repurposed TB drugs demonstrate in recent clinical trials like, NIX-TB, ZeNIX and TB PRACTECAL considerable treatment success, shorten treatment duration and reduce toxicity. Their optimal use is threatened by the rapid occurrence and spread of strains, resistant to new drugs. Children benefit only very slowly from the progress.

**Summary:**

There is notable progress in improved diagnosis and treatment of drug-resistant TB, but complicated by the COVID-19 pandemic the majority of TB patients worldwide don’t have (yet) access to the advances.

## INTRODUCTION

**FIGURE 1:**
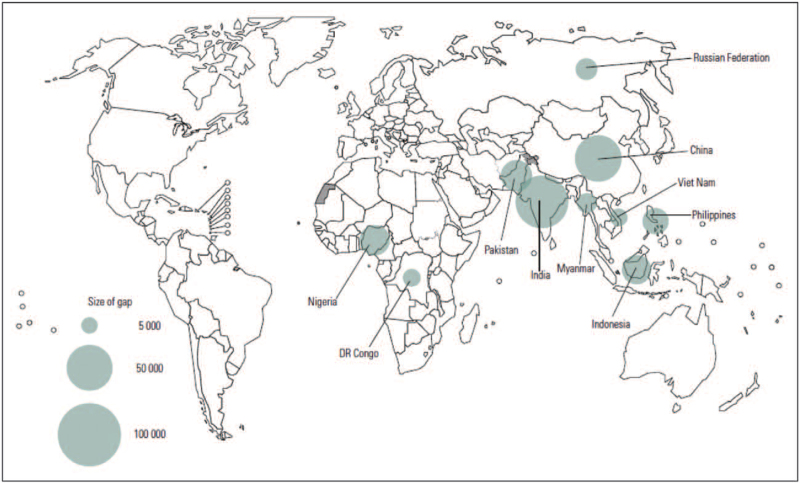
Ten countries with largest gaps between number of patients started on treatment for multidrug-resistant/rifampicin- resistant-tuberculosis and best estimates of multidrug-resistant/rifampicin-resistant-tuberculosis incidence 2019 (order of countries from biggest to lowest gap: India, Pakistan, Nigeria, Indonesia, Philippines, Russian Federation, Myanmar, DR Congo, Vietnam). Adapted from [3].

Drug-resistant tuberculosis (DR-TB) has been a concern since the introduction of the first anti-TB chemotherapeutic drugs [1] and continues to threaten global efforts to curb an otherwise curable disease. The World Health Organisation (WHO) reports that in 2020, only 150329 patients with multidrug-resistant (MDR) or rifampicin-resistant TB were enrolled on treatment, a third of the estimated burden [2,3], highlighting a capacity gap for either diagnosing or treating DR-TB - a gap that comes at the cost of continued transmission of drug-resistant strains (Fig. 1; Table 1). Compared with susceptible TB, DR-TB presents a lower probability for treatment success and yet requires more resources for treatment. The treatment success rate for MDR/rifampicin-resis- tant TB was reported as 59% globally in 2020, compared with 86% for new and relapse drug susceptible TB [2]. The diagnostic landscape for TB has seen significant developments in the last decade, most notable of which have led to the widespread adoption of molecular diagnostic technologies. The treatment landscape, although lagging behind diagnosis, has seen the abandonment of toxic drugs including injectables and shortening of regimens for DR-TB and potentially drug susceptible TB [4]. 

**Box 1:**
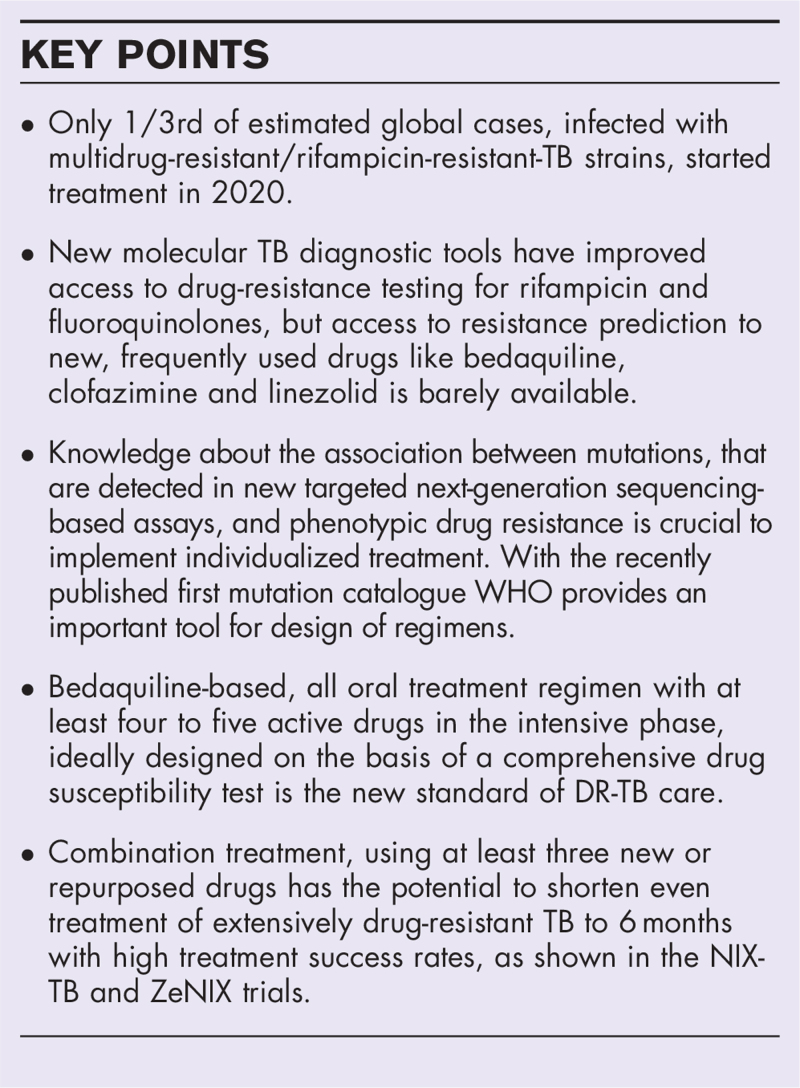
no caption available

**Table 1:** Key epidemiologic indicators of drug-resistant- tuberculosis 2021 [2]

Indicator	Proportion/number
Global estimated cases of RR-TB 2019^a^	500000 (95% CI 400 000–535 000)
Proportion of new TB cases with RR-TB^a^	3.3%
Proportion of retreatment cases with RR-TB^a^	17.7%
Proportion of new, pulmonary TB, smear positive tested for Rifampicin resistance	69%
MDR/RR cases tested for fluoroquinolone resistance	78 000
Lab-confirmed MDR/RR cases	158000
MDR/RR cases started treatment	150 000
Lab-confirmed pre-XDR-TB/XDR-TB cases	26000
pre-XDR-TB/XDR-TB cases started treatment	22 000
Global treatment success for MDR-TB	59%

CI, confidence interval; MDR, multidrug-resistant; RR, rifampicin-resistant; TB, tuberculosis; XDR, extensively drug-resistant.

a Data from Global tuberculosis report 2020 [3].

The advent of the COVID-19 pandemic in 2020 has allowed the application of lessons and infrastructure from TB care and prevention, but also offers an opportunity for TB care and prevention to learn from rapid technology deployment and public health interventions introduced to curb COVID-19 [5^▪^]. This review article will provide an brief overview of the most recent evidence to assist in the diagnosis and treatment of DR-TB.

## DIAGNOSIS OF *MYCOBACTERIUM TUBERCULOSIS* DRUG RESISTANCE

Diagnosing TB is one of the most challenging aspects of the cascade of care. Pulmonary active DR-TB is identified in primary diagnostics by routine clinical workup with focus-centred sampling (sputum, tracheobronchial lavage for active respiratory TB) in adult patients or stool, respectively gastric fluid samples in children. A recently published simple protocol enhances the diagnostic performance in stool, in particular in children, where respiratory specimen sampling remains very challenging [6^▪^].

In cases with suspicion of extrapulmonary TB (EPTB) sampling from the target organ is essential. Nonsputum based tests are required in particular for diagnosis of EPTB, people living with HIV and in children. The next-generation lipoarabinomannan (LAM) test are showing improved performance over original LAM tests [7,8], but don’t detect TB drug resistance. WHO guidelines on TB diagnosis, updated in 2021, summarize the current evidence and recommendations on TB diagnosis [9].

WHO has recommended the use of rapid molecular tests to diagnose MDR-TB since 2015 [10]. State- of-the-art molecular platforms screen for the presence of *Mycobacterium tuberculosis* complex (Mtbc) DNA and resistance to rifampicin in a single assay. Xpert MTB/RIF (Cepheid, Sunnyvale, CA, USA) got a WHO endorsement in 2014 [11,12]. The assay revolutionized the time-to-result screening and confirmation of rifampicin-resistant TB. The following steps in the evolution of the molecular tests involved lowering the detection limit (Xpert MTB/ RIF Ultra) and evaluating additional resistance-associated genes [13], also improving TB diagnostics in children [14]. Line probe assays to detect TB drug resistance are currently being replaced by gene arrays, respectively, next-generation sequencing (NGS)-based methods such as Deeplex Myc-TB (Genoscreen, Lille, France) [15,16^▪▪^]. Further, there is a broader offer of automated rapid real-time PCR assays for the direct detection of TB and drug resistance [17]. Nevertheless, drug resistance to new and repurposed drugs threatens recent advances in DR- TB treatment and appropriate resistance testing to those drugs is widely unavailable [18,19]. WHO and the Foundation for Innovative New Diagnostics developed a set of target product profiles to guide the development of new TB diagnostic tools [20,21]. The patient-focused approach should prompt for fast clinical evaluation and early laboratory diagnostic testing. All patients with TB should be risk- assessed for drug resistance based on epidemiological data and history [22]. The diagnostic workup involves point-of-care molecular testing to screen for TB and rifampicin resistance. If rifampicin-resistant Mtbc is detected, add-on modular testing for first-line and second-line drugs are recommended. The new Xpert MTB/XDR screens for mutations leading to resistance to isoniazid, fluorquinolones, ethionamide and aminoglycosides and can offer a first, reliable assessment of the susceptibility profile of the infectious agent [23^▪^]. On a global scale, confirmatory testing using mycobacterial liquid culture, bacterial identification and phenotypic drug-susceptibility testing (pDST) can be provided in national and supranational reference laboratories mainly. State-of-the-art pDST uses the WHO critical concentration drug proportion method, which can be performed in semiautomated instruments such as MGIT 960 (BD, Franklin Lakes, New Jersey, USA) [24]. Alternative testing methods using broth microdilution are being implemented in various supranational laboratories, which generate quantitative susceptibility profiles, especially for antibiotics that entered the therapeutic armamentarium only recently such as bedaquiline, delamanid and pretomanid [25^▪^].

The EUCAST reference method of minimum inhibitory concentration (MIC) determination for Mtbc is the broth microdilution method in Middlebrook 7H9–10% OADC (Oleic Albumin Dextrose Catalase) medium. The MIC, expressed in mg/l, is the lowest concentration that inhibits visible growth [26]. Currently, epidemiological cut-offs for therapeutically used drugs are being established to generate data for clinical breakpoints. In addition to pDST, sequencing based resistance detection is being launched in various centres. WHO has recently published a catalogue of Mtbc mutations and their association with phenotypic drug resistance. The catalogue provides a reference standard for interpreting mutations conferring resistance to all first-line and a variety of second-line drugs. The report summarizes the analysis of over 38 000 isolates with matched data on whole genome sequencing and pDST testing from over 40 countries for 13 anti-Mtbc drugs. It lists over 17 000 mutations, their frequency and association with resistance, including methods used, mutations identified and summaries of significant findings for each drug [27^▪▪^]. NGS diagnostics coupled with bioinformatic pipelines to analyse genetic data can predict TB drug resistance precisely and will be the new standard for resistance detection and therapeutic recommendations [28^▪^,^29^].

## PREVENTION

There is still inadequate evidence on the impact of TB preventive treatment for contacts of patients with MDR/rifampicin-resistant TB [30]. Results from the three randomized trials TB CHAMP, V-QUIN and PHOENIx MDR-TB are eagerly awaited [31].

## TREATMENT OF DRUG-RESISTANT TUBERCULOSIS

Treatment of DR-TB is undergoing a very dynamic period with many improvements for patients under TB care. A recent systematic review and meta-analysis on ototoxicity of kanamycin, amikacin and cap- reomycin demonstrated, that in 18 studies from 10 countries 40.6% (95% confidence interval 32.8–66.6%) of patient suffered persistent hearing loss after exposure to the second-line injectables [32]. This figure alone underlines the extreme importance of recent change to all oral DR-TB treatment regimen. WHO issued such guidance first in a rapid communication from 2018 [33], which was based on the results of a large individual patient data analysis [34]. This individual patient data analysis also led to the regrouping of drugs in groups A, B and C for the design of longer regimen, based on certainty of evidence for effectiveness and safety (Table 2). New guidelines in 2019 then suggested new treatment options: an all oral longer (18–20 month) regimen, an injectable containing shorter (9–12 month) regimen and an all oral shorter (9–12 months) regimen used under operational research conditions [35]. In 2019 evidence from South Africa demonstrated advantages ofa 9–12 months all oral, bedaquiline containing regimen [36]. WHO made the recommendation by the end 2019 to replace 4–6 months of injectable (mostly amikacin, as kanamy- cin and capreomycin were not recommended anymore), with 6 months bedaquiline in an all oral shorter regimen (4–6 months Bdq-Lfx-Eto-E-Z-Hh- Cfz/5 Lfx-Cfz-Z-E)[37]. The most recent guidelines by WHO consolidated this recommendation [38]. Amikacin and streptomycin remain group C drugs. Their use is only recommended in a longer, individ-ualized regimen for MDR/rifampicin-resistant-TB, which should contain at least four likely effective drugs from group A and B during the intensive phase, and three effective drugs after stopping beda- quiline after about 6 months. In settings with sufficient resources, individualized therapy, designed using targeted NGS-based drug susceptibility testing, will probably soon replace above mentioned programmatic approaches [28^▪^]. Biomarkers to determine optimal duration of treatment are urgently needed. Recent results, using transcrip- tomic signatures generate hope for progress in this field [39^▪^,40^▪^].

**Table 2:** Group A, B and C drugs: design and steps of longer treatment regimen for drug-resistant tuberculosis according WHO classification (initially at least four active drugs, if Bdq is stopped after 6 months, continue at least three active drugs)

Group	Medicine	Abbreviation
A Include all three drugs	Levofloxacin or moxifloxacin Bedaquiline Linezolid	Lfx Mfx Bdq Lzd
B Add one or both	Clofazimine Cycloserin or terizidone	Cfz Cs Trd
C Add to complete the regimen, or when drugs from group A or B cannot be used	Ethambutol Delamanid Pyrazinamide Imipenem-Cilastin or meropenem Amikacin or (streptomycin) Ethionamide or prothionamide P-amino salicylic acid	E Dlm Z Ipm-Cln Mpm Am (S) Eto Pto PAS

Adapted from WHO operational handbook on tuberculosis; Module 4; drug-resistant tuberculosis [38].

The recommendation against the use of kana- mycin and capreomycin and classification of streptomycin and amikacin as a group C drug made it necessary to redefine extensively drug-resistant TB (XDR-TB). Previously defined as MDR-TB with additional resistance to a fluoroquinolone and a second line injectable, XDR-TB was redefined in 2020 as resistance against at least a later generation fluoroquinolone and one other group A drug in addition to rifampicin and isoniazid [41].

The NIX-TB trial tested the new nitro-imidazole pretomanid in combination with bedaquiline and linezolid 1200 mg (BPaL regimen) for 26 weeks in patients with XDR-TB (old definition) and patients with inappropriate treatment response during treatment of MDR-TB [42^▪▪^]. This single arm, open-label study *(n* *=* 109) documented an unfavourable outcome in only 10% of patients 6 months after end of treatment. But 81% showed signs of peripheral neuropathy and 48% myelotoxicity, which had to be actively managed by dose reductions and treatment interruptions. Based on those results the ZeNIX trial tested the same regimen with different dosages or duration of linezolid. The dose adaptations showed less toxicity and similar efficacy. A linezolid dose of 1200 mg/6 months showed 93% favorable outcome; 1200 mg/2 months 89%, 600 mg/6 months 91% and 600 mg/2 months 84%, respectively. Linezolid 600 mg/6 months and 600 mg/2 month showed neuropathy in 24 and 13%, myelotoxicity in 2 and 7%. Bedaquiline was given as 200 mg daily for 8 weeks and then 100 mg daily for 26 weeks [43^▪▪^]. The BPaL regimen therefore provides a first real treatment option for patients with highly drug resistant TB with a duration of only 6 months. WHO recommended the BPaL regimen under operational research conditions for patients with MDR-TB and fluorquinolone resistance and a maximum previous exposure duration of 2 weeks for bedaquiline or linezolid [38].

TB PRACTECAL is an open label, randomized controlled trial with an adaptive multistage design, which is dedicated to shorten DR-TB treatment. The trial results, first presented at the conference of the International Union of TB and Lung Disease 2021, document the potency of drugs used in the NIX and ZeNIX trial. In TB PRACTECAL stage II a regimen with bedaquiline 400 mg daily for 2 weeks, then 200 mg 3 times per week, pretomanid 200 mg daily, moxifloxacin 400 mg daily and linezolid 600 mg daily for 16 weeks, then 300 mg daily for 8 weeks with a total treatment duration of 24 weeks showed a favourable outcome in 88.7% in the modified intention-to-treat population compared with 51.5% in the standard of care group. The standard of care was adapted at sites to the guidelines changes during the recent years. The safety of the investigational regimen was much improved compared with standard of care with 19.5 versus 58.9% severe adverse events of grade 3 or higher [44^▪▪^]. The results of these trials open a new chapter in the care for patients DR-TB and will likely allow to dramatically shorten DR-TB treatment, provide much higher treatment success rates and manageable toxicity.

On the contrary, in one of the very few phase III randomized controlled trials providing evidence in DR-TB care, the nitroimidazole delamanid did not shorten the time to culture conversion compared with placebo, added to an optimized background regimen, but showed a good safety profile [45]. The STREAM II trial is a phase III randomized controlled trial, where bedaquiline for 40 weeks replaces kana- mycin for 16 weeks using the short course ‘Bangladesh’ regimen [46]. Results are expected in 2022.

Cardiotoxicity is an important safety aspect of bedaquiline and delamanid use. The DELIBERATE trial evaluated both drugs individually or in combination in patients with MDR/rifampicin-resistant- TB, receiving a background regimen, disallowing clofazimine and replacing moxifloxacin with levofloxacin. Antiretroviral treatment was based on dolutegravir. Mean change in QTc was 12.3, 8.6 and 20.7 ms for bedaquiline, delamanid and their combined use, with no grade 3 or 4 adverse QTc related events or deaths among the 84 (31 HIV-positive) patients randomized in the 24 weeks study period. The authors concluded that the combination of delamanid and bedaquiline in patients with normal baseline QTc interval was safe to use [47].

A major challenge remains the access of children, with an estimated incidence of 25 000–32000 cases annually, to recent achievements in MDR/ rifampicin-resistant TB care [48]. Recent clinical trial data and review of evidence led in August 2021 to the recommendation by WHO, that bedaquiline and delamanid may be used in children of all ages [49,50].

To develop new regimens or even a pan-TB regimen, which can treat drug-susceptible and DR- TB similarly, new drugs are required. Telacebec (Q203), an imidazopyridine amide which inhibits the mycobacterial cytochrome bc1 complex, is the third new class of drugs with human antituberculous activity, after bedaquiline, a diarylquinoline and the nitroimidazoles delamanid and pretoma- nid. A phase II, early bactericidal activity study, comparing telacebec to a standard regimen, has shown the potential of the new drug to be tested further in clinical trials [51].

Several other compounds and drugs are under preclinical and clinical development. The webpage of the working group of new anti-TB drugs gives a good, uptodate overview (https://new-tbdrugs.org). An additional important topic is the development of host-directed therapies, with the goal to reduce post-TB lung disease, mortality and improve functional long-term outcomes [52,53]. A recent phase II randomized controlled trial, comparing everolimus, CC 11050, auranofin and ergocholecalciferol suggested a possible protective effect of everolimus and CC 11050 on lung function (FEV1) [54^▪▪^].

## CONCLUSION

Several new developments in the diagnosis and treatment of drug-resistant TB have occurred in the last decade, and the pipeline is not empty. Rapid molecular diagnostic assays that detect both TB and drug resistance have been made widely available, with rapid expansion to their capability. Other molecular technologies such as NGS are becoming more widely available and are supported by WHO- endorsed reference catalogue and bioinformatic systems. As work continues in the search of more accessible gold standards for diagnosing DR-TB, culture-based technologies based in central-level laboratories remain relevant for TB and resistance detection and setting standards. Any routine workup for TB should now include a molecular test for rifampicin resistance, with add-on tests for further resistance if rifampicin resistance is detected. The versatility of newer molecular platforms allows for multiplexing with other tests. Various treatment options have been explored recently, with a trajectory towards shorter, injection-free and hopefully safer regimens. Paradoxically, efficacy of shorter regimens is being demonstrated even for highly resistant strains. However, the optimal preventive treatment for MDR/rifampicin- resistant TB has not garnered a wide consensus.

Many challenges in the diagnosis and treatment of DR-TB remain. Notably, while the new WHO definitions for XDR-TB will no doubt spur advocacy and technological developments in the area, they are a challenge to implement in most settings due to lack of infrastructure for resistance testing for new and repurposed drugs [55]. New technologies cannot be implemented rapidly enough to those in need [18]. The COVID-19 pandemic has not only exacerbated the inequities in health access, but has interfered with the allocation of resources for TB care and prevention. Nevertheless, there are opportunities to piggyback on the resources such as multiplexing on diagnostic platforms. The rapidly changing treatment guidelines and multiple possible regimens make local adaptation to country settings difficult as drug-forecasting and capacity-building have to be accelerated in the context of limited resources. If indeed, the newer regimens improve treatment completion and overall outcomes these trade-offs may well be worth it.

## Acknowledgements

*None.*

### Financial support and sponsorship

*No funding was received in context of this review article.*

### Conflicts of interest

*There are no conflicts of interest.*

## REFERENCES AND RECOMMENDED READING

Papers of particular interest, published within the annual period of review, have been highlighted as:

▪ of special interest
▪▪ of outstanding interest
